# [Corrigendum] All-*trans* retinoic acid alters the expression of the tight junction proteins Claudin-1 and -4 and epidermal barrier function-associated genes in the epidermis

**DOI:** 10.3892/ijmm.2024.5399

**Published:** 2024-07-08

**Authors:** Jing Li, Qianying Li, Songmei Geng

Int J Mol Med 43: 1789-1805, 2019; DOI: 10.3892/ijmm.2019.4098

Following the publication of the above article, the authors contacted the Editorial Office to explain that they had identified a pair of duplicate images in the control (Vehicle) group of mouse images in Fig. 1A on p. 1792. Specifically, the same image (corresponding correctly to the 'Day 5' experiment) was inadvertently chosen to represent the cutaneous manifestations of mice in the Vehicle group on 'Day 3' and 'Day 5' in Fig. 1A. This error arose as a consequence of repetitive application and duplication procedures within the image set, resulting in the inadvertent reuse of the same photo. Additionally, due to minimal alterations observed in the skin condition of mice from the control group following treatment, each mouse exhibited a similar appearance; this similarity further contributed to the delayed identification of this error during the paper revision stage. Consequently, this duplication of the same image was made as a result of insufficient scrutiny.

The revised version of Fig. 1, showing the correct image for the 'Day 3' experiment in Fig. 1A, is shown on the next page. The authors can confirm that the error associated with the assembly of this figure did not have any significant impact on either the results or the conclusions reported in this study, and all the authors agree with the publication of this Corrigendum. The authors are grateful to the Editor of *International Journal of Molecular Medicine* for allowing them the opportunity to publish this; furthermore, they apologize to the readership of the Journal for any inconvenience caused.

## Figures and Tables

**Figure 1:**
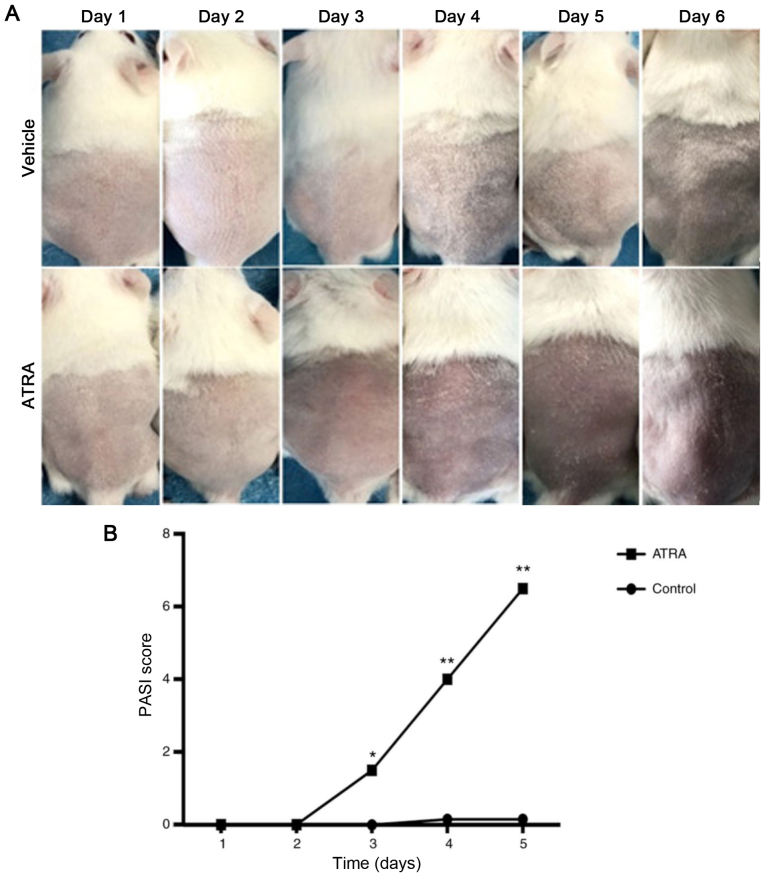
Effect of ATRA on morphological changes and PASI score on the back skin of mice. (A) After 5 days of treatment, no obvious alterations were detected in the control skin. Circumscribed erythema occurred after 3 days of ATRA application and gradually expanded, presenting as fine flat scales covering the surface of the erythema and peaking at 5 days. (B) The PASI scoring system was used to assess the severity of inflammation on the back skin of the mice. ^*^P<0.05, ^**^P<0.01 vs. respective control. PASI, Psoriasis Area and Severity Index; ATRA, all-trans retinoic acid.

